# NAAG Peptidase Inhibitors Act via mGluR3: Animal Models of Memory, Alzheimer’s, and Ethanol Intoxication

**DOI:** 10.1007/s11064-017-2181-4

**Published:** 2017-03-11

**Authors:** Rafal T. Olszewski, Karolina J. Janczura, Tomasz Bzdega, Elise K. Der, Faustino Venzor, Brennen O’Rourke, Timothy J. Hark, Kirsten E. Craddock, Shankar Balasubramanian, Charbel Moussa, Joseph H. Neale

**Affiliations:** 1 Department of Biology, Georgetown University, 37th and O Sts., N.W., Washington, D.C. 20057-1225 USA; 2 Department of Neuroscience, Georgetown University, Washington, D.C. 20057 USA

**Keywords:** *N*-Acetylaspartylglutamate, NAAG, Memory, mGluR3 knockout mice, Alzheimer’s mice, Ethanol intoxication, Glutamate carboxypeptidase II, GCPII

## Abstract

Glutamate carboxypeptidase II (GCPII) inactivates the peptide neurotransmitter *N*-acetylaspartylglutamate (NAAG) following synaptic release. Inhibitors of GCPII increase extracellular NAAG levels and are efficacious in animal models of clinical disorders via NAAG activation of a group II metabotropic glutamate receptor. mGluR2 and mGluR3 knock-out (ko) mice were used to test the hypothesis that mGluR3 mediates the activity of GCPII inhibitors ZJ43 and 2-PMPA in animal models of memory and memory loss. Short- (1.5 h) and long- (24 h) term novel object recognition tests were used to assess memory. Treatment with ZJ43 or 2-PMPA prior to acquisition trials increased long-term memory in mGluR2, but not mGluR3, ko mice. Nine month-old triple transgenic Alzheimer’s disease model mice exhibited impaired short-term novel object recognition memory that was rescued by treatment with a NAAG peptidase inhibitor. NAAG peptidase inhibitors and the group II mGluR agonist, LY354740, reversed the short-term memory deficit induced by acute ethanol administration in wild type mice. 2-PMPA also moderated the effect of ethanol on short-term memory in mGluR2 ko mice but failed to do so in mGluR3 ko mice. LY354740 and ZJ43 blocked ethanol-induced motor activation. Both GCPII inhibitors and LY354740 also significantly moderated the loss of motor coordination induced by 2.1 g/kg ethanol treatment. These data support the conclusion that inhibitors of glutamate carboxypeptidase II are efficacious in object recognition models of normal memory and memory deficits via an mGluR3 mediated process, actions that could have widespread clinical applications.

## Introduction

*N*-Acetylaspartylglutamate (NAAG), a prevalent and widely distributed peptide co-transmitter, is inactivated by glutamate carboxypeptidase II (GCPII) following synaptic release [1]. Inhibitors of GCPII [2, 3] are effective in animal models of several clinical conditions [reviewed in 4–6]. These inhibitors enhance long-term memory in the 24 h delay novel object recognition test [7], improve memory in an animal model of multiple sclerosis [8], rescue behaviors and short-term memory impairment in animal models of schizophrenia [9–11]. Consistent with these results, mice that are null mutant for GCPII demonstrate full memory in the 24 h delay novel object recognition test, while their heterozygous littermates and wild type C57Bl mice exhibit no significant recall in this test of long-term memory [7]. GCPII inhibitors also are analgesic in models of inflammatory and neuropathic pain [12–14] and reduce the effects of traumatic brain injury [15] while GCPII knockout (ko) mice are protected from peripheral neuropathy and ischemic and traumatic brain injury [16–18].

NAAG reduces transmitter release from neurons and synaptosomes via a group II mGluR receptor [19, 20]. Inhibitors of GCPII elevate extracellular levels of NAAG and also reduce the release of glutamate and other transmitters [13, 21, 22]. These neurochemical actions of the peptidase inhibitors and their positive effects in animal models are blocked by the group II mGluR antagonist LY341495. While a substantial body of data supports the conclusion that the peptide activates a group II metabotropic receptor [4, 20], highly purified NAAG fails to activate mGluR2 or mGluR3 receptors expressed in transfected cells [23, 24], results suggesting that some reports of NAAG activation of a group II mGluR were due to the presence of low levels of residual glutamate (≤0.5% [25]) in commercially available NAAG. In contrast, data from other studies preclude the conclusion that the NAAG activity is due to this level of contaminating glutamate [20]. Consistent with an action of NAAG at mGluR3, NAAG peptidase inhibition blocks the motor activation effects of phencyclidine in mGluR2, but not mGluR3, ko mice [10]. In those studies where the effects of NAAG or NAAG peptidase inhibition have not been shown to be blocked by a group II mGluR antagonist, it is possible that NAAG is acting as an antagonist at a subclass of NMDA receptors [26].

In order to further test the hypothesis that a group II mGluR, specifically mGluR3, mediates the procognitive efficacy of GCPII inhibitors, these compounds were tested across a series of animal models that included short- and long-term novel object memory, Alzheimer’s disease, and acute alcohol intoxication, using a group II antagonist in wild type mice and testing mice that are null mutant for mGluR2 and mGluR3.

## Methods

### Animals

The experimental protocols used in this research were approved by the Georgetown University Animal Care and Use Committee and are consistent with guidelines of the US National Institutes of Health. Efforts were made to reduce animal suffering and to minimize the number of animals used. Adult male C57BL/6NCr mice from the National Cancer Institute, Frederick Research Center were tested once at 2–4 months of age and used for all studies except those involving the knock out or transgenic mice. The mGluR2 and mGluR3 ko mice (knock out R1 cell lines into C58Bl/6 mice and backcrossed into ICR[CD1] mice) [27] were provided by Eli Lilly Pharmaceuticals and tested twice at 3–5 months of age in a novel object recognition study. Wildtype littermates for these ko mouse colonies were not available. There are no published studies that compare the performances of these ko mice with their wild type littermates in the novel object recognition test. The triple-transgenic mouse model (3xTg line) that expresses three genes associated with familial Alzheimer’s disease, namely APP_Swe_, PS1_M146V_, and tau_P301L_ [28] were from Jackson Labs (Strain: B6;129-Psen1 Tg(APPSwe,tauP301L)1Lfa/Mmjax; genetic background: (129 × 1/SvJ x 129S1/Sv)F1-Kitl<+>; JAX MMRRC Stock# 034830) and tested at 2–9 months of age in the short-term novel object recognition test. Mice were housed 5 to a cage and maintained on a 12:12 h light–dark cycle with food and water available ad libitum. Behavioral testing was performed during the light cycle between 10 am and 4 pm.

### Drugs

The GCPII/NAAG peptidase inhibitor ZJ43 (ZJ43 (*N*-[[[(1*S*)-1-carboxy-3-methylbutyl]amino]carbonyl]-l-glutamic acid) was synthesized as previously described [9] and provided by Alan Kozikowski. The GCPII inhibitor 2- ((2-(phosphonomethyl)pentane-1, 5-dioic acid) [3, 29]) was from Reagents4Research, LLC (Hangzhou, CN). LY341495, a selective group II mGluR antagonist [30], and LY354740, a heterotropic group II mGluR agonist [31], were from Tocris Cookson Ltd. (Bristol, UK). All compounds were dissolved in saline and pH was adjusted to 7.4 prior to i.p. injection. Ethanol (2.1 g/l, ip) was given as a concentration of 20% v/v in saline. Doses of ZJ43, 2-PMPA, LY341495, LY354740 and ethanol were based on data from published and preliminary studies.

### Novel Object Recognition Test

Novel object recognition is a validated and widely used test for assessing recognition memory [32–35], including in studies of aging [36, 37] and Alzheimer’s disease mouse models [38, 39]. Individual mice (3–4 month old) were placed in a 22 × 32 × 30 cm testing chamber with beige walls for a 5 min habituation interval followed by i.p. injection with saline, 2-PMPA (50 mg/kg) or ZJ43 (150 mg/kg), with or without LY341495, and returned to home cage. Thirty minutes later, mice were placed in the testing chamber for 10 min with two identical objects (acquisition session). Mice were returned to home cages and 1.5 h (short-term memory) or 24 h (long-term memory) later were returned to testing chamber in the presence of one of the original objects and one novel object (recognition session) for 10 min. Wild type mice exhibit short-term but not long-term memory in this test [7, 10]. The original objects consisted of two smooth surfaced weighted red cylinders 7 cm high × 4 cm diameter at base. The novel object consisted of a blue, 7 cm high × 5 cm diameter (base) round pyramid. The acquisition and recognition sessions were video recorded and the time mice spent exploring each object was assessed by an observer who was blinded to drug treatment and genotype. The chambers and objects were cleaned with ethanol between trials. Exploratory behavior was defined as sniffing, touching and directing attention to the object. In preliminary studies, naïve mice exhibited no significant preference for the red cylinder or the blue pyramid. Exploration time (Table 1) is expressed as the mean ± the standard error of the mean (SEM). For the acquisition session, the recognition index was calculated as (time exploring one of the objects/the time exploring both objects) × 100. For the recognition session, the RI was calculated as (time exploring the novel object/the time exploring both the familiar and novel object) × 100.

**Table 1:** Exploration time data from short-term (Figs. 2, 3, and 4) and long-term novel object recognition (Fig. 1)

Group	N	Acquisition session (s)		Recognition session (s)	
		FO (1)	FO (2)	NO	FO (2)
m2ko and m3ko mice—long-term memory—Fig. 1					
m2ko—saline	11	13.7 (0.8)	13.6 (0.9)	8.2 (1.2)	8.2 (1.1)
m2ko—PMPA	12	15.3 (0.9)	15.0 (0.9)	16.5 (1.1)	6.2 (0.9)
m2ko—ZJ43	11	14.6 (1.2)	15.1 (1.2)	10.5 (1.3)	5.2 (0.7)
m3ko—saline	11	18.3 (0.9)	18.7 (0.5)	12.3 (1.2)	12.0 (1.2)
m3ko—PMPA	11	17.8 (1.1)	17.6 (0.9)	13.6 (2.2)	13.0 (2.3)
m3ko—ZJ43	12	17.7 (0.9)	18.1 (0.8)	13.7 (1.3)	12.6 (1.0)
AD mice—Fig. 2—short -term memory—Fig. 2					
8 Weeks saline	15	11.4 (1.0)	12.5 (1.1)	22 (3.5)	12.6 (2.7)
5 Months saline	12	4.5 (0.7)	5.1 (0.8)	6.6 (0.9)	6.8 (0.7)
9 Months saline	10	22.3 (3.3)	21.3 (3.9)	26.6 (4.4)	25.9 (3.5)
9 Months PMPA	10	19.9 (2.5)	20.8 (3.5)	45.2 (5.4)	27.4 (6.0)
Ethanol treated mice—short-term memory—Figs. 3 and 4					
S-S	10	20.4 (1.9)	21.4 (2.5)	25.2 (2.0)	11.9 (1.6)
S-EtOH	11	25.2 (1.9)	27.0 (2.1)	21.8 (2.0)	18.5 (1.8)
ZJ43-EtOH	6	14.2 (2.0)	16.0 (2.4)	10.7 (2.6)	5.33 (1.2)
2-PMPA-EtOH	6	11.5 (0.7)	11.0 (0.8)	8.3 (1.3)	2.8 (0.9)
LY40 (2)-EtOH	12	3.1 (0.4)	2.8 (0.4)	4 (1.0)	4.2 (1.4)
LY40 (5)-EtOH	12	2.9 (0.4)	3.2 (0.5)	6.6 (1.0)	2.6 (0.3)
LY40 (10)-EtOH	7	4.14 (1.0)	4.2 (1.0)	8.0 (2.6)	2.9 (0.6)
mGluR2ko-S	11	45.5 (4.9)	45.7 (7.4)	60.7 (9.4)	31.0 (5.5)
mGluR2ko-EtOH	12	12.7 (2.7)	13.3 (2.5)	23.6 (4.6)	20.2 (3.5)
mGluR2ko-PMPA + EtOH	10	7.0 (1.5)	7.3 (1.4)	14.5 (2.3)	9.7 (1.5)
mGluR3ko-S	9	20.8 (2.5)	17.7 (3.2)	21.3 (3.5)	12.7 (2.9)
mGluR3ko-EtOH	9	8.0 (2.4)	7.1 (2.6)	14.3 (3.3)	15.1 (3.0)
mGluR3ko-PMPA + EtOH	10	5.4 (1.0)	6.3 (1.4)	12.8 (3.4)	12.9 (3.6)

**Fig. 1:**
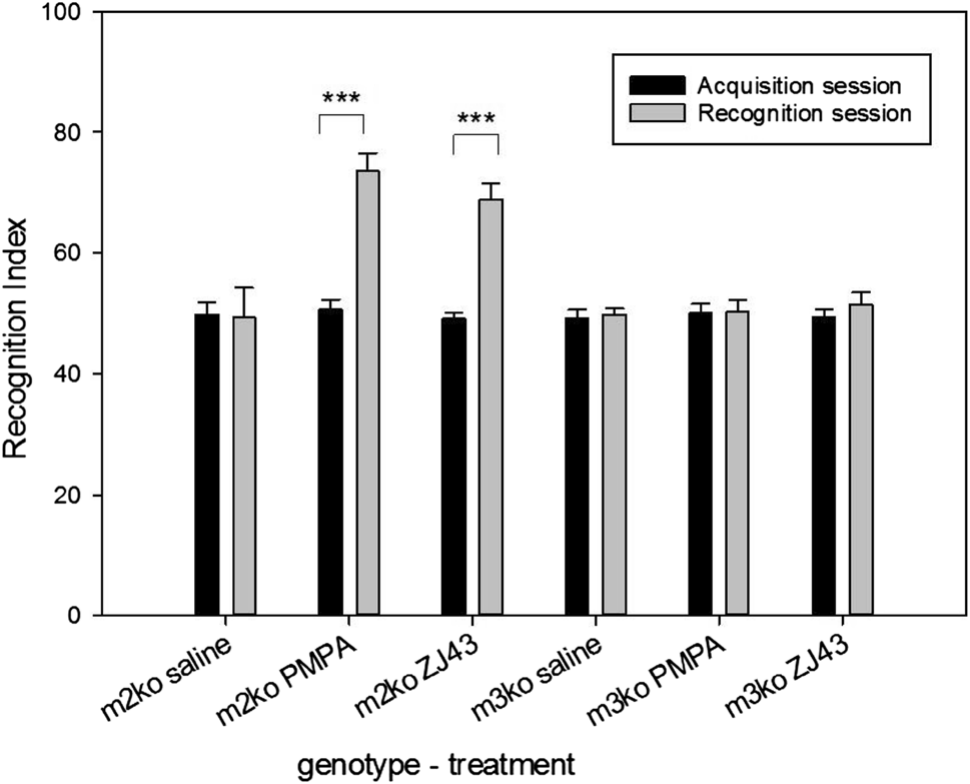
Long-term novel object recognition memory test in mGluR2 and mGluR3 KO mice. In this and the following novel object recognition figures: for the acquisition session, the recognition index (RI) was calculated as (time exploring one of the objects/the time exploring both objects) × 100. For the recognition session, the RI was calculated as (time exploring the novel object/the time exploring both the familiar and novel object) × 100. During the acquisition phase, each group of mice explored each of the two identical objects about the same amount of time (recognition index ~50). During the recognition phase 24 h later, the mGluR2 KO mice (m2ko) treated with saline explored the novel and familiar object similar amounts of time while those treated with 2-PMPA (100 mg/kg) or ZJ43 (150 mg/kg) explored the novel object twice as often as the original object (recognition index ~70), while the NAAG peptidase inhibitors had no procognitive effect in the mGluR3 ko mice (m3ko). m2ko/saline, n = 11; m2ko/PMPA, n = 12; m2ko/ZJ43, n = 11; m3ko/saline, n = 11; m3ko/PMPA, n = 11; m3ko/ZJ43, n = 12. *p < 0.05, **p < 0.01, ***p < 0.001 for comparison between acquisition session and recognition session within treatment group in Figs. 1, 2 and 3

**Fig. 2:**
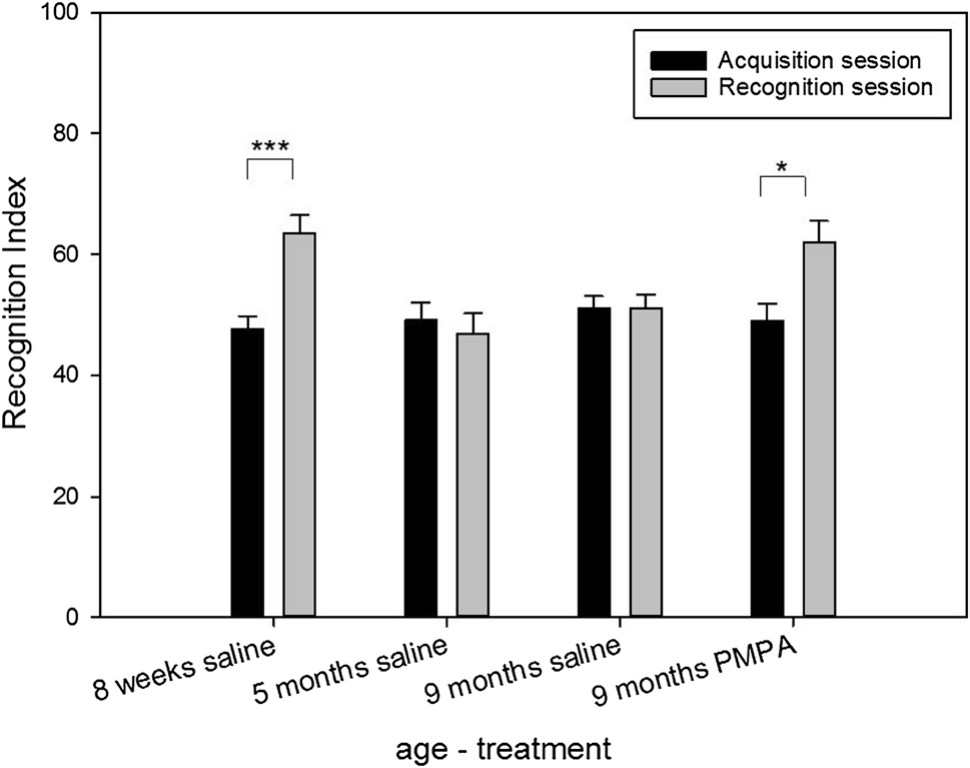
Short-term novel object recognition memory test in triple transgenic Amice. Eight week old AD mice explored the novel object significantly more than the familiar object while the 9 month old AD mice failed to discriminate between the novel and familiar object. 2-PMPA (100 mg/kg) restored the ability of the older mice to discriminate between the novel and familiar object. 8 week, n = 15; 9 mos/saline, n = 10; 9 mos/PMPA, n = 10

**Fig. 3:**
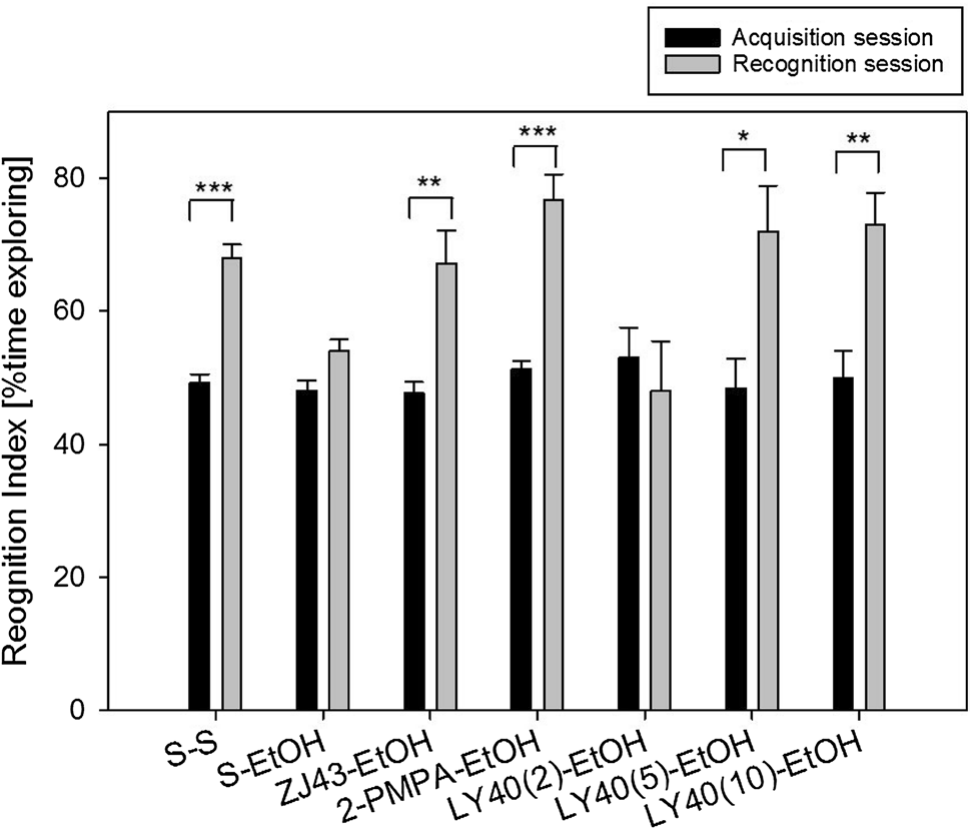
Ethanol impairment of short-term novel object recognition is reversed by NAAG peptidase inhibitors and the group II mGluR agonist LY354740. Mice treated with two injections of saline before the acquisition phase of the test, explored the novel object significantly more than the familiar object 1.5 h later (recognition index ~70). Ethanol (2.1 g/kg) blocked discrimination of the novel object in the retention session. Pretreatment with ZJ43 (150 mg/kg) and 2-PMPA (100 mg/kg) reversed the cognitive deficits induced by ethanol. Pretreatment with LY354740(LY40) dose dependently reversed the effects of ethanol. S-S, n = 10; S-EtOH, n = 11; ZJ43-EtOH, n = 6; 2-PMPA-EtOH, n = 6; LY40(2)-EtOH, n = 12; LY40(5)-EtOH, n = 12; LY40(10)-EtOH, n = 7

*FO* time spend exploring familiar object, *NO* time spent exploring novel object, *LY40* LY354740 (group II agonist). Exploration time for each group expressed as the mean ± the standard error of the mean (SEM). mGluR ko were tested for long-term memory 24 h after acquisition session (Fig. 1). mGluR ko, control mice and treated mice were tested for short-term memory 1.5 h after acquisition session (Figs. 2, 3, and4)

**Fig. 4:**
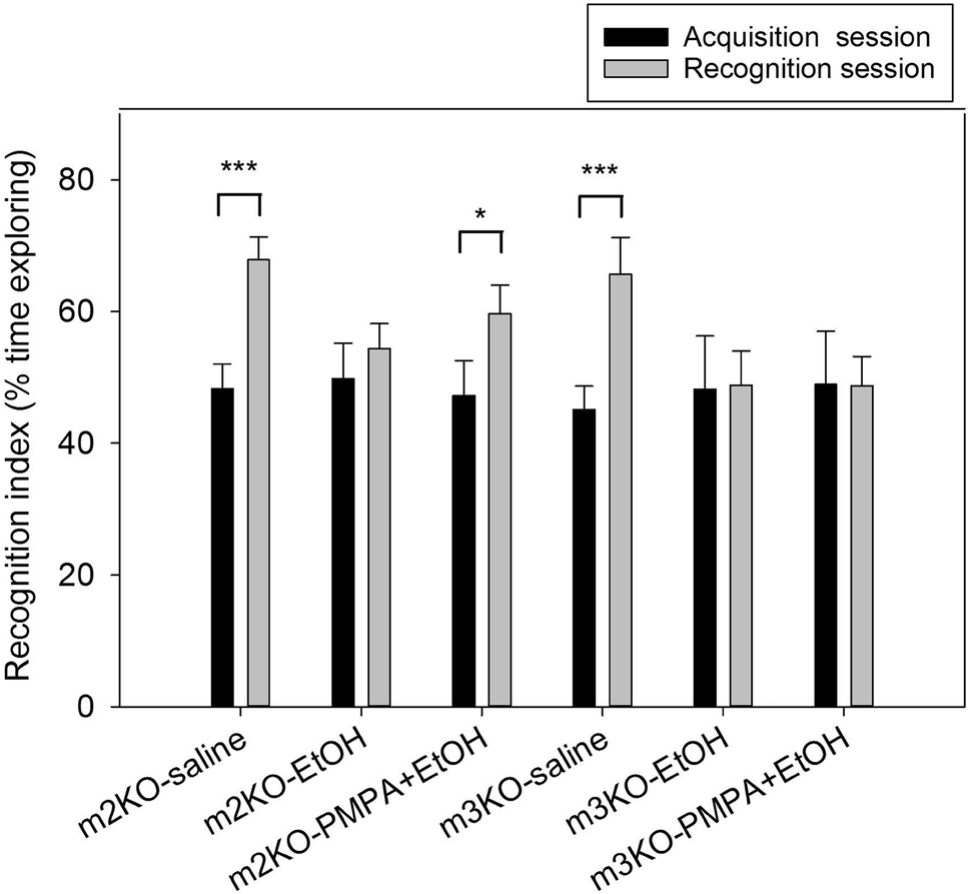
mGluR3 mediates the procognitive effects of NAAG peptidase inhibition in the ethanol treatment model. mGluR2 (m2KO) and mGluR3 KO (m3KO) mice exhibited short-term (1.5 h) memory in the novel object memory test. Mice of both strains failed to discriminate between the novel and familiar object when treated with 2.1 g/kg ethanol prior to the acquisition trial. 2-PMPA (100 mg/kg) partially reversed the effect of ethanol in mGluR2 but not mGluR3 mice. mGluR2ko-S, n = 11; mGluR2ko-EtOH, n = 12; mGluR2ko-PMPA + EtOH, n = 10; mGluR3ko-S, n = 9; mGluR3ko-EtOH, n = 9; mGluR3ko-PMPA + EtOH, n = 10

To study the effects of ethanol on short-term memory, mice were placed the testing chamber for a 5 min habituation interval followed by injection with saline, ZJ43 (150 mg/kg), 2-PMPA (100 mg/kg) or LY354740 (2, 5, 10 mg/kg) and returned to home cage. Thirty minutes later mice were injected with ethanol (2.1 g/kg, i.p.) and returned to their home cage for 10 min after which they were placed in a testing chamber for 10 min with two identical objects (acquisition session). Mice were returned to home cages and 1.5 h later were placed back into the testing chamber for 10 min in the presence of one of the original objects and one novel object (recognition session).

### Open Field Motor Activation Test

High doses of ethanol induces increases in motor activity (40) and loss of coordination (41) in mice. In the present study, mice were habituated to an open field chamber (Med Associates, St., Albans, Vermont, ENV-515 43 × 43 cm, with infrared beams and detectors) for 30 min prior to i.p. injection with saline, ZJ43 (150 mg/kg)with or without LY341495 (3 mg/kg), or with LY354740, returned to the chamber for 15 min, and injected (i.p.) with 2.1 g/kg of ethanol. Locomotor activity was then recorded as distance travelled during 10 min in the open field chamber.

### Rotorod Test

The rotorod was used to assess motor coordination and balance. Mice were injected (i.p.) with saline, ZJ43 (150 g/kg), 2-PMPA (10, 50, 100 mg/kg) with or without LY341495, or with LY354740 (10 mg/kg), returned to their home cage in the testing room and 15 min later were injected (i.p.) with 2.1 g/kg ethanol. Forty-five minutes later mice were placed on the drum (70 mm dia) facing away from the direction of the rotation so they can walk forward at constant speed (4 rpm) for 10 s of habituation. The drum was then accelerated over 3 min, from 0 to 40 rpm (with cut off time = 3 min) and the latency to fall from the drum was recorded. Each animal was tested three times with 15 min between trials.

### Statistical Analysis

For the novel object recognition test, the time spent exploring each object was analyzed by two-way repeated measures ANOVA, with session as within-subject factor and treatment as a between-subject factor. Discrimination ratio data were analyzed by one-way ANOVA followed by Student–Newman–Keuls post-hoc test. Motor activation data and rotorod data were analyzed with GLM ANOVA followed by post-hoc Tukey test.

## Results

### Total Exploration Times

The time exploring individual objects during acquisition trials and recognition trials for each treatment group are presented in Table 1. Within treatment groups, there was a wide range of total exploration times in the acquisition and retention sessions. Drug treatments combined with ethanol tended to result in less attention to the objects during the acquisition trials. This was particularly evident in the LY354740 with ethanol (2.1 g/kg) treatment groups but also observed in the ko mice treated with ethanol. An additional anomaly is the substantial difference in the total exploration times of mGluR2 ko/saline treated mice versus the mGluR2ko/ethanol treated mice. Despite these differences between groups, there are clear and significant drug effects in the recognition sessions. The reliability of the novel object recognition data is supported by the fact that, despite differences in the total exploration times among the treatment groups, both objects are nearly equally attended during the acquisition session across all groups and that the recognition data fall clearly into two categories: nearly equal attention to both objects (failed memory) or significantly greater attention to the novel object than familiar object (memory). There were no apparent effects of 2-PMPA or ZJ43 on mean exploration times during the acquisition trials relative to saline treated mice.

### NAAG Peptidase Inhibitors ZJ43 and 2-PMPA are Procognitive **for Long-term Memory** in mGluR2 and but not mGluR3 Knockout Mice

Mice lacking functional mGluR2, like wild type C57BL mice [7], explored the two similar objects to the same extent during the acquisition trial and failed to discriminate the between the novel and familiar objects when tested one day later (Fig. 1). Also as in wild type mice [7], both NAAG peptidase inhibitors, ZJ43 (150 mg/kg, i.p.) and 2-PMPA (50 mg/kg, i.p.) increased (p < 0.001) exploration of the novel object when the drugs were given prior to the acquisition trial on day 1.

mGluR3 knockout mice also spent similar amounts of time exploring the two identical objects during the acquisition trial and the novel and familiar object during the retention trial. However, neither ZJ43 nor 2-PMPA was procognitive in these mice based on their failure to elicit greater exploration of the novel object in the retention trial.

### NAAG Peptidase Inhibitor 2-PMPA is Procognitive for Short-Term Memory in Triple Transgenic Alzheimer’s Mice

The triple-transgenic (3xTg line) mice express three genes associated with familial Alzheimer’s disease, APP_Swe_, PS1_M146V_, and tau_P301L_. The same mice were tested at 2, 5 and 9 months of age in the novel object test of short-term memory. At 2 months of age, these mice demonstrated short-term memory while they failed in this test at 5 and 9 months of age (Fig. 2). The 9-month old mice had a high level of exploratory behavior in both the acquisition and retention trials (Table 1). Treatment with 100 mg/kg of 2-PMPA prior to the acquisition phase reversed this memory deficit (p = 0.05) in the 9-month old mice as demonstrated by their level of exploration of the novel object relative to the familiar object.

### Acute Ethanol Intoxication and Short-Term Memory

C576BL/6 mice (3–4 month old) treated with saline prior to the acquisition trial explored the two identical objects about the same amount of time (Fig. 3) and when presented with one novel object and one familiar object 1.5 h later, they explore the novel object about twice as frequently as the familiar object [11]. In this study, there was a main effect of treatment and session (F_(6,59)_ = 4.51, p < 0.01, F_(1,59)_ = 199.32, p < 0.001) and a treatment x session interaction (F_(6,59)_ = 3.73, p < 0.01). Mice treated with ethanol (2.1 g/kg, i.p.) prior to the acquisition trial also explored the two objects about the same amount of time. However, in contrast to the saline treated control mice, the ethanol treated mice explored the novel and familial objects equally in the retention trial suggesting a failure to recall or recognize the familiar object relative to the novel one. The NAAG peptidase inhibitors ZJ43 (150 mg/kg), 2-PMPA (100 mg/kg) and the type 2/3 metabotropic glutamate receptor agonist LY354740 (10 mg/kg) reverse the effects of ethanol on novel object recognition (p < 0.01, p < 0.001 and p < 0.01 respectively). A low dose of the type 2/3 metabotropic glutamate receptor antagonist LY341495 (2 mg/kg) failed to block the effects of 2-PMPA on ethanol treatment in this assay. When given alone to control mice prior to acquisition, 2 and 3 mg/kg of LY341495 reduced memory on the retention trial and could not be used to confirm the role of NAAG at the group II metabotropic glutamate receptors in this study. Mice given 1.7 g/kg (i.p.) ethanol did not exhibit a significant loss of short-term memory in this assay (data not shown).

In the absence of confirmation that the mGluR mediated the efficacy of NAAG peptidase inhibition in the ethanol intoxication model, the study was repeated using mGluR2 and mGluR3 ko mice treated with saline or 2.1 mg/kg (i.p.) ethanol (Fig. 4). Saline treated mice of both strains showed a significant level of recognition of the novel object and this short-term memory was blocked by pretreatment with ethanol. mGluR2 ko mice pretreated with 2-PMPA (100 mg/kg) prior to ethanol administration demonstrated significant memory (p < 0.05) in the recognition trials while 2-PMPA was without a significant effect in the ethanol treated mGluR3 ko mice.

### NAAG Peptidase Inhibitors Block Ethanol-Induced Motor Activation

**Fig. 5:**
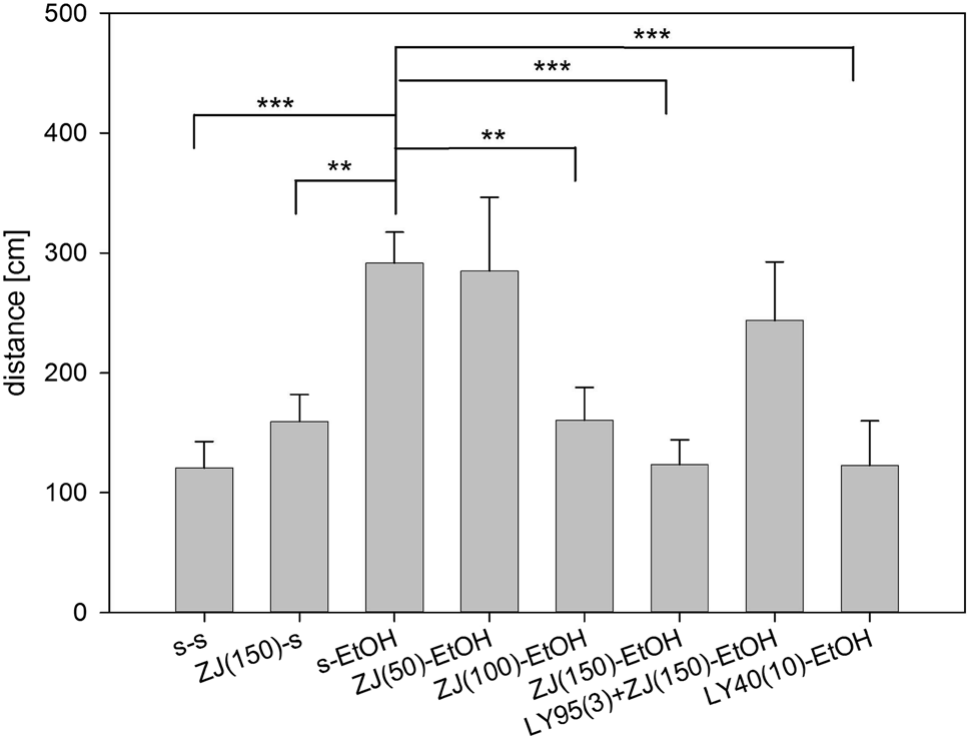
Ethanol-induced motor activation reversed by ZJ43 and group II agonist LY354740. Ethanol (2.1 g/kg, i.p.) increases motor activation in open field test. Pretreatment with ZJ43 (50, 100 and 150 mg/kg, i.p.) dose dependently reduced motor activation during the 10 min interval immediately following ethanol administration. The group II metabotropic glutamate receptor agonist LY354740 (10 mg/kg) was similarly effective in moderating the effects of ethanol. The group II metabotropic glutamate receptor antagonist LY341495 reversed the effect of ZJ43 (150 mg/kg) on ethanol-induced motor activation. S-saline. ZJ = ZJ43, LY95 = LY341495, LY40 = LY354740. N: s/s (11), ZJ150/s, ZJ100/Et-OH, LY95 + ZJ150/Et-OH (9), s/Et-OH (20), ZJ50/Et-OH, ZJ150/Et-OH, LY40/Et-OH (10). *p < 0.05, **p < 0.01, ***p < 0.001

Ethanol (2.1 g/kg, i.p.) induced a prompt increase (p < 0.001) in motor activation in mice placed in an open field chamber to which they previously had been habituated (Fig. 5). There was a main effect of drug (saline, ZJ43 or LY doses, F_(6,80)_ = 4.679, p < 0.001) and treatment (saline or ethanol, F_(1,80)_ = 15.503, p < 0.001). Pretreatment with ZJ43 (50, 100 and 150 mg/kg, i.p.) dose dependently reduced motor activation during the 10 min interval immediately following ethanol administration (50 mg/kg, p < 0.01 and 150 mg/kg, p < 0.001). The group II metabotropic glutamate receptor agonist LY354740 (10 mg/kg) reversed the effects of ethanol (p < 0.001). To confirm the role of NAAG and a type 2 or 3 metabotropic glutamate receptor in mediating the effects of NAAG peptidase inhibition, ZJ43 (150 mg/kg) was co-administered with the group II antagonist LY341495 (3 mg/kg). The antagonist reversed the effect of 150 mg/kg ZJ43 (p < 0.05). The group II mGluR agonist LY354740 (10 mg/kg, i.p.) also blocked the ethanol effect.

### Rotorod Test of Ethanol-Induced Loss of Motor Coordination or Balance

**Fig. 6:**
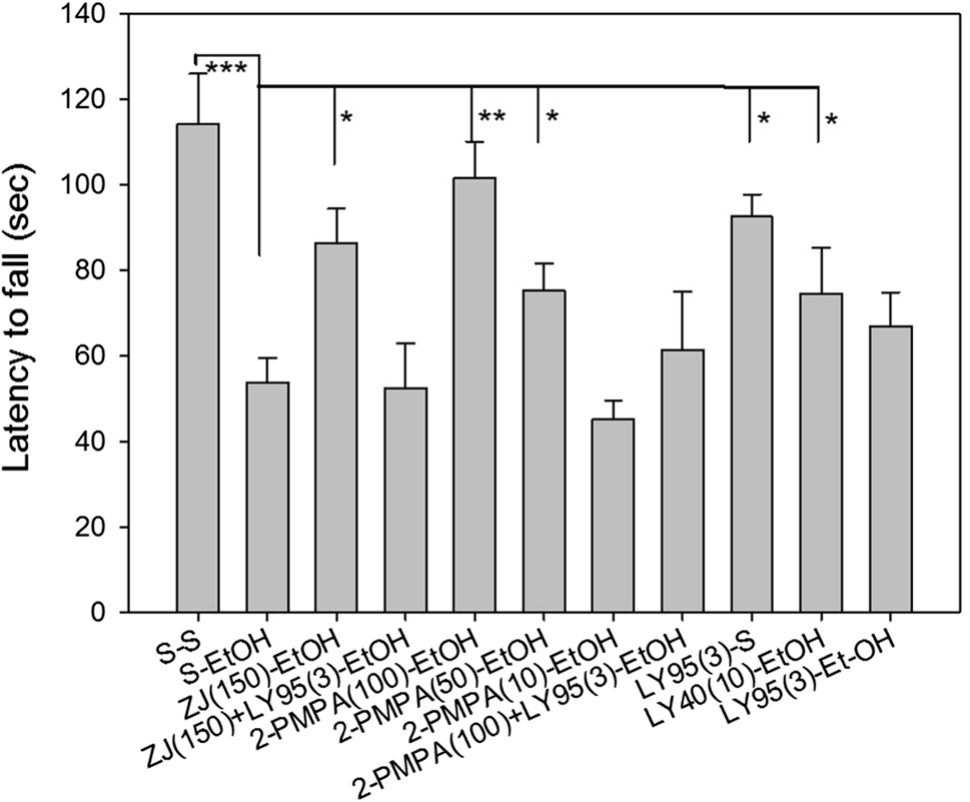
ZJ43 and 2-PMPA moderate ethanol-induced loss of balance on Rotarod Test. **a** Ethanol (2.1 g/kg, i.p.) significantly reduced latency to fall relative to saline treated mice (p < 0.001). 2-PMPA reversed the effect in dose dependent manner (p < 0.01 for 100 mg/kg and p < 0.05 for 50 mg/kg comparing with ethanol group). ZJ43 (150 mg/kg) and LY354740 (10 mg/kg) also reduced the effects of 2.1 g/kg ethanol on latency to fall (both p < 0.05). The group II antagonist LY341495 (3 mg/kg) blocked the effect of 2-PMPA (100 mg/kg) and ZJ43 (150 mg/kg), both at p < 0.05 versus 2-PMPA and ZJ43. S-saline, LY95 = LY341495, LY40 = LY354740. All groups are compared individually for statistical significance versus the saline-ethanol group. N: s/s(10), s/EtOH(12), ZJ43-EtOH(12), ZJ43 + LY95/EtOH(17), 2-PMPA100-EtOH(8), 2-PMPA50-EtOH(8), 2-PMPA10-EtOH(8), 2-PMPA100 + LY95(3)EtOH(8), LY95/s(10), LY40/EtOH(8), LY95/EtOH(10)

In the rotorod test, mice given with ethanol (2.5 g/kg, i.p.). There was a significant effect of drug (F_(8,100)_ = 3.458, p < 0.01) and treatment (F_(1,100)_ = 22.409, p < 0.001). Ethanol treatment produced a 55% reduction in latency to fall from the rotorod relative to saline treated mice (p < 0.001) (Fig. 6). ZJ43 (150 mg/kg, i.p.) blocked this effect of ethanol. 2-PMPA (10, 50 and 100 mg/kg) blocked the effect of 2.1 g/kg ethanol in a dose-dependent manner (p < 0.01 for 100 mg/kg and p < 0.05 for 50 mg/kg comparing with ethanol group). The effects of ZJ43 and 2-PMPA were reversed by the group II mGluR antagonist LY341495 (3 mg/kg, p < 0.05) while this antagonist alone had no significant effect on the ethanol-induced loss of motor coordination. The group II mGluR agonist LY354740 (LY40, 10 mg/kg) also reduced the effects of 2.1 g/kg ethanol on latency to fall (both p < 0.05).

## Discussion

### mGluR3 Mediates Procognitive Efficacy of NAAG Peptidase Inhibition

The purpose of this study was to further test the procognitive effects of NAAG peptidase inhibition across models of normal memory and in clinically relevant models of cognitive deficits. In parallel, the goal was to test the hypothesis that these procognitive effects are mediated by NAAG activation of the group II metabotropic glutamate receptor, mGluR3. While many strains of mice are reported to demonstrate short-term (1.5 h) memory in the novel object recognition test, there are no reports of mice exhibiting long-term (24 h) memory in this test. Similarly, the mGluR2 and mGluR3 ko mice in this study showed significant memory in the short-term test (Fig. 4) and the absence of memory of the familiar object in the long-term memory test (Fig. 1). In previous studies, NAAG peptidase inhibition reversed short-term memory deficits elicited by a low dose of the NMDA antagonist MK801 [11] and had procognitive activity in the 24 h delay test [7]. Both of these actions were blocked by the group II mGluR antagonist LY341495, consistent with a series of studies that support the conclusion that the peptide mediates group II mGluR activation [20, 42, 43]. The finding that the procognitive effects of 2-PMPA and ZJ43 in the 24 h delay novel object recognition test were observed in mGluR2 but not mGluR3 ko mice (Fig. 1), supports the conclusion that mGluR3 is the group II receptor mediating these procognitive actions. This conclusion is further strengthened by the efficacy of 2-PMPA in partially reversing ethanol-induced cognitive impairment of short-term memory in mGluR2 but not mGluR3 ko mice (Fig. 4). A similar result in support of a role for mGluR3 in the efficacy of NAAG peptidase inhibition was observed in a mouse model of schizophrenia [10]. Central to the conclusion that these effects of the peptidase inhibitors are mediated by the activation of mGluR3 by elevated levels of synaptically released NAAG, rather than by the drugs directly, are the reports that high levels (100 UM) of ZJ43 and 2-PMPA do not activate group II receptors in vitro [12, 44].

These data on the mGluR3 mediated procognitive actions of NAAG peptidase inhibition also are consistent with the broader hypothesis that this receptor plays a more general role in memory formation or retrieval. Supporting this view, mGluR3 ko mice showed deficits in working memory when tested in T- and Y mazes and polymorphisms in mGlur3 are associated with cognitive deficits in schizophrenia patients [45, 46].

### NAAG as mGluR3 Agonist

A rigorous study of the effect of purified NAAG on hippocampal slices and cells transfected with mGluR2 or mGluR3 [24] found no evidence of the peptide activating a group II receptor and suggested that some prior reports of NAAG activation of these receptors could have been due to a previous report that commercial NAAG was contaminated with 0.3–0.4% glutamate [25]. While this glutamate effect cannot be discounted for some studies, it does not seem consistent with other results [42]. For example, NAAG and glutamate dose responses for group II mGluR activation differed by no more than threefold when tested against cerebellar astrocytes, cells that expressed mGluR3 message but little if any mGluR2 [43]. The failure of NAAG to activate group II receptors in transfected cells [23, 24] further contrasts the report that NAAG is several orders of magnitude more potent than glutamate in reducing transmitter release from spinal cord synaptosomes [20], an action that was blocked by the group II antagonist LY341495 and the mGluR3 selective antagonist beta-NAAG [47]. Very high levels of NAAG and NAAG peptidase activity are expressed in spinal cord and spinal sensory neurons [48, 49] and peptidase inhibition moderated the effect of spinal cord trauma [50].

At the moment, there are not sufficient data to resolve the apparent conflict among the data on the failure of NAAG to activate mGluR3 in transfected cells and the studies presented here and elsewhere [4, 7, 10, 11, 13, 20, 42] in which the effects of NAAG and NAAG peptidase inhibition are blocked by group II mGluR antagonists and are absent in mGluR3 ko mice. One possibility is that the mechanism of expression or dimerization of mGluR3 following its transfection into non-neuronal or -glial cell lines differs from that in vivo, in spinal cord synaptosomes or in cultured astrocytes.

### NAAG Peptidase Inhibition in Alzheimer’s Disease Model Mice

The APP_Swe_, PS1_M146V_, and tau_P301L_ transgenic mouse line [28] captures both the beta-amyloid and Tau neuropathology found in Alzheimer’s disease [51] and thus represents one of the most widely studied animal models of this disorder. Age-dependent behavioral changes have been characterized in this mouse line including deficits in novel object recognition and attention [52–55]. The novel object recognition test also has been used to characterize other transgenic animal models of Alzheimer’s disease [35, 56] and recognition memory for novel objects serves as a marker for clinical diagnosis of this disorder [57]. This test is particularly useful in distinguishing cognitive loss in normal aging versus loss in Alzheimer’s disease model mice inasmuch as different strains of mice retain short-term memory in the novel object recognition test well beyond 9 months of age even while other cognitive functions are declining [37] In the present study, short-term novel object recognition was observed in the transgenic mice at 2 months but not at 5 and 9 months of age. Consistent with prior reports of procognitive effects of NAAG peptidase inhibition [7, 8, 11], 2-PMPA significantly improved performance on this task in the 9-month old triple mutant Alzheimer’s disease mice. While normal 9- month old wild type mice were not tested in this study, a substantial literature demonstrates mice of different strains, including non-transgenic colony mates of triple transgenic Alzheimer’s mice do not exhibit a decline in short-term novel object recognition as late as 22 months of age [36, 38, 39, 58–60]. Thus, while the behavioral test presented here does not speak to cognitive deficits associated with normal aging in mice, it will be interesting to determine if NAAG peptidase inhibitors are procognitive in other behavioral tests in which normal mice demonstrate aging-related cognitive deficits. In a similar study, we found that ZJ43 also reversed the cognitive deficit of aged triple transgenic mice in the novel object recognition test and the efficacy of ZJ43 was reversed by the group II mGluR antagonist (Olszewski and Neale, work in progress).

### Broader Impact of NAAG Peptidase Inhibition in Cognition

The observations that NAAG peptidase inhibition and deletion of GCPII are procognitive in control conditions, where the mice have not been cognitively challenged via a drug treatment ([7] and Fig. 1), suggest that the procognitive actions of these peptidase inhibitors in animal models of clinical conditions, such as Alzheimer’s disease (Fig. 2), schizophrenia [8, 11] and ethanol intoxication (Fig. 3), might not be specifically reversing the neurochemical processes that underlie these clinical models but rather be generally procognitive. However, while the conditions that induce these models are clearly different, they can be linked by the common element of increases in glutamate release and NAAG peptidase inhibitors have been consistently shown to reduce synaptic release of glutamate [13, 14, 21, 22, 61–63]. In any case, the procognitive efficacy of NAAG peptidase inhibition in long-term novel object recognition, demonstrate that NAAG’s role on cognition is not limited to deficits induce by excess glutamate release.

In human studies, treatment with growth hormone-releasing hormone increased NAAG levels in the prefrontal cortex and improved performance of subjects exhibiting mild cognitive impairment [64]. Similarly, NAAG levels in the hippocampus positively correlated with cognitive functioning in multiple sclerosis patients [8].

### Other Group II mGluR Ligands in Cognition Studies

The mGluR2/3 agonist LY379268 had a procognitive effect in the novel object recognition test [65] while heterotropic group II mGluR agonists and an mGluR2 positive allosteric modulator have procognitive effects in other behavioral tests [66, 67]. Yet in other studies, agonists including the LY354740 impair rather than enhance attention and working memory [68–70]. Interpretation of these reports is complicated by the use of agonists and antagonists that interact with both mGluR2 and mGluR3 in vitro and in vivo [27, 30, 31, 71]. In studies using ko mice, some heterotrophic group II agonists have been shown to work via mGluR2 rather than mGluR3 [69, 72, 73]. This leads to the possibility that the contrasting effects of heterotrophic group II agonists on cognition may reflect differences in the behavioral tests that were used or their differential actions on mGluR2 and mGluR3 receptors. Evidence that the procognitive effects of NAAG peptidase inhibition could be specific to the type of memory being tested comes from the report that 100 mg/kg of 2-PMPA in mice does not affect long-term memory in the step through passive avoidance test or spatial working memory in the Y maze [74].

### NAAG Peptidase Inhibition and Ethanol Induced Cognitive Deficits in Short-Term Novel Object Recognition

NAAG peptidase inhibition consistently reversed the cognitive impairment induced by ethanol in short-term novel object recognition (Fig. 3 and4). We previously reported that NAAG peptidase inhibition alone had no effects on cognition in this test [11]. The data in Fig. 4 clearly demonstrate that the efficacy of these inhibitors in this short-term memory test require mGluR3. While the effect of 2-PMPA in the mGluR2 ko mice does not reflect a complete reversal of the action of ethanol, the recognition index for this group if mice is significantly different from the acquisition session.

### Ethanol, Glutamate and Group II mGluR

Glutamate appears to have a central role in drug addiction and alcoholism [75]. The efficacy of NAAG peptidase inhibition in reversing the effects of ethanol treatment (Figs. 3, 4, 5, 6) is consistent with the view that group II receptors, particularly mGluR3, are among the more promising targets for the development of drugs to treat alcohol addiction [76–78] Heterotropic group II glutamate receptor agonists reduce drug seeking, conditioned place preference and stress-induced reinstatement in animal models of alcohol addiction [76, 79, 80]. The agonist LY379268 also blocks the effects of alcohol on discriminative stimulus testing [81], alcohol self-administration and reinstatement [82]. Additionally, epistatic effects of genetic variants of the mGluR3 and COMT genes have been associated with hippocampal volume in alcohol-dependent patients but not in controls [83]. The activity of NAAG peptidase inhibition in the present study might again be related to the efficacy of the peptide in reducing release of glutamate inasmuch as ethanol induces glutamate release in the nucleus accumbens, hippocampus and posterior ventral tegmental area, brain regions known to mediate some of the clinical effects of alcohol consumption [84–86].

The stimulant effects of alcohol in adolescents [40] are modeled in mice by open field motor activation induced immediately after administration [41]. In the present study using mice that previously had been habituated to the open field chamber, ethanol (2.1 g/kg) induced an increase in open field activity relative to control mice over the first 10 min minutes after injection (Fig. 5). Pretreatment of mice with NAAG peptidase inhibitors reduced this initial motor activation. Such a result might be taken to indicate that NAAG peptidase inhibitors have a sedative effect as they similarly block motor activation induced by PCP and *d*-amphetamine [9–11, 87]. However, these inhibitors, given alone, do not affect motor activity in control mice habituated or unhabituated to open field conditions [11].

Data from animal models suggest that mGluR3 agonists might be useful in treatment of ethanol addiction [76–78]. The data presented here demonstrate that drugs that elevate NAAG levels also moderate motor activation, cognitive and balance effects of ethanol intoxication and support the conclusion that mGluR3 mediates these effects. The breadth of actions of NAAG peptidase inhibitors in these studies suggests that they may be affecting a central mechanism underlying ethanol-induced intoxication.

## Conclusion

NAAG peptidase inhibition improves cognition in the novel object recognition tests and reduces the cognitive and motor deficits induced by ethanol via a cellular mechanism that involves activation of mGluR3. The GCPII inhibitor 2-PMPA also reverses the cognitive deficit observed in an animal model of Alzheimer’s disease. These and other data support the conclusion that GCPII is a significant target for the development of procognitive drugs.

## Abbreviations

NAAG: *N*-Acetylaspartylglutamate
mGluR: Metabotropic glutamate receptor
mGluR2: Metabotropic glutamate receptor type 2
mGluR3: Metabotropic glutamate receptor type 3
ko: Knockout
GCPII: Glutamate carboxypeptidase II
